# Physical activity, obesity and mortality: does pattern of physical activity have stronger epidemiological associations?

**DOI:** 10.1186/s12889-017-4806-6

**Published:** 2017-10-05

**Authors:** Adrian E. Bauman, Anne C. Grunseit, Vegar Rangul, Berit L. Heitmann

**Affiliations:** 1 Prevention Research Collaboration, School of Public Health, University of Sydney, Level 6, Charles Perkins Centre, Johns Hopkins Drive, Sydney, NSW 2006 Australia; 2 HUNT Research Centre, Faculty of Medicine, Department of Public health and General practice, NTNU – Norwegian University of Science and Technology, Levanger, Norway; 3 The Boden Institute of Obesity, Nutrition, Exercise & Eating Disorders, University of Sydney, Sydney, NSW 2006 Australia; 4 Research Unit for Dietary Studies at the Parker Institute and Institute of Preventive Medicine, Frederiksberg and Bispebjerg Hospital, Copenhagen, Denmark; 5 Institute of Public Health, University of Southern Denmark, Odense, Denmark; 6 Copenhagen Center for Preventive Medicine, Glostrup Hospital, Copenhagen Capital Region, Denmark

**Keywords:** Waist circumference, Hip circumference, Cardiovascular disease

## Abstract

**Background:**

Most studies of physical activity (PA) epidemiology use behaviour measured at a single time-point. We examined whether ‘PA patterns’ (consistently low, consistently high or inconsistent PA levels over time) showed different epidemiological relationships for anthropometric and mortality outcomes, compared to single time-point measure of PA.

**Methods:**

Data were the Danish MONICA (MONItoring Trends and Determinants in CArdiovascular Disease) study over three waves 1982–3 (time 1), 1987–8 (time 2) and 1993–4 (time 3). Associations between leisure time single time-point PA levels at time 1 and time 3, and sport and active travel at times 1 and 2 with BMI, waist, hip circumference and mortality (death from coronary heart disease (CHD) and cardiovascular disease (CVD)) were compared to ‘PA patterns’ spanning multiple time points. PA pattern classified participants’ PA as either 1) inactive or low PA at both time points; 2) moderate level PA at time 1 and high activity at time 3; or 3) a ‘mixed PA pattern’ indicating a varying levels of activity over time. Similarly, sport and active travel were also classified as indicating stable low, stable high and mixed patterns.

**Results:**

The moderately and highly active groups for PA at times 1 and 3 had up to 1.7 cm lower increase in waist circumference compared with the inactive/low active group. Across ‘PA patterns’, ‘active maintainers’ had a 2.0 cm lower waist circumference than ‘inactive/low maintainers’. Waist circumference was inversely related to sport but not active travel. CHD risk did not vary by activity levels at time 1, but was reduced significantly by 43% for high PA at time 3 (vs ‘inactive’ group) and among ‘active maintainers’ (vs ‘inactive/low maintainers’) by 62%. ‘Sport pattern’ showed stronger reductions in mortality for cardiovascular disease and CHD deaths among sport maintainers, than the single time point measures.

**Conclusions:**

PA patterns demonstrated a stronger association with a number of anthropometric and mortality outcomes than the single time-point measures. Operationalising PA as a sustained behavioural pattern may address some of the known under-estimation of risk for poor health in PA self-report measurements and better reflect exposure for epidemiological analysis of risk of health outcomes.

**Electronic supplementary material:**

The online version of this article (10.1186/s12889-017-4806-6) contains supplementary material, which is available to authorized users.

## Background

Six decades of epidemiological studies have identified the health consequences of physical inactivity, with clear and consistent evidence for a relationship with all-cause mortality and cardiovascular disease (CVD) but more inconsistently with weight gain and fat distribution [1]. For example, studies have suggested that associations between physical activity (PA) and weight gain, overweight and obesity are weak or inconsistent, and that weight development and overweight may predict physical inactivity rather than the reverse direction [2]. On the other hand, the relationship of PA with mortality shows large and consistent effect sizes. The average meta-analytic risk reduces by 33% for all-cause mortality for the regularly active compared to the inactive [3], with slightly smaller estimates from more recent meta-analyses [4]. For moderate intensity activities only, the relative risk reduction is 19–24% [5]. Data are similar for CVD mortality risk reductions [6]. In summary, meta-analytic reviews suggest a 25–30% risk reduction of fatal outcomes in those who are physically active compared to the inactive.

However, a methodological limitation in this field is that most PA epidemiologic studies examine activity behaviour or measured fitness at a single point in time in relation to subsequent health outcomes or mortality. The frequency of single-point-in-time exposure measure mortality studies from an earlier (1975 and 2000) and later (2014–2016) period is quantified in Additional File 1. This sample of PA and mortality epidemiological studies indicates that 91% of those published earlier period, 93% in the later period use a single time-point PA exposure. Eight studies in each of the periods have more than one PA measurement prior to the occurrence of the outcome. As PA is a complex and changing behaviour, the epidemiologic relationships between PA and outcomes may be better characterized by assessing the pattern of the behaviour over time. Unlike behaviours such as tobacco smoking (that are reasonably stable over time), behaviours such as PA or diet may vary continuously over time [7, 8]. Tracking coefficients have been estimated for adults to fall within the low to moderate range (0.35 to 0.65) but appear to be lower over longer time intervals (for example, 0.25–0.29) [9]. If the exposure measure varies temporally to a substantial degree, then the risk estimates between PA and health outcomes may be underestimated, due to misclassification bias. Further, taking a life course perspective through multiple measures at different time points may reveal additional benefits of PA by testing the effect of consistency. Therefore, for PA it may be better to examine changes in activity, or even a longer-term patterns of PA in relation to health outcomes.

A larger number of studies have examined change in PA in relation to changes in weight or waist circumference and suggest that a change in PA may be an important determinant for preventing weight gain. For example previous research has shown lower gains in waist circumference over six years amongst those in a constant active group for leisure time PA, and for men for occupational PA [10]; lower weight and waist gain among men and especially women who increased their PA over five years compared to those with a static activity level [11]; those maintaining high levels compared with those maintaining low levels over 20 years [12]; and lower weight gain among women who increased bicycling over 16 years [13]. Of the many studies examining mortality outcomes, a few have considered change in PA or cardiorespiratory fitness and subsequent risk of death [14, 15]. However, these epidemiological studies have not examined whether a ‘pattern of the behaviour of being active’ over time may be more reflective of a lifetime behavioural pattern and show a stronger association with health outcomes specifically when compared to a single exposure measure. One study of walking/running did conduct both single-point and change in PA analyses, but restricted the former to only those with a consistent ‘PA pattern’ whilst using baseline PA as the exposure and hence no comparison between the two analyses was made [16].

This paper examines ‘patterns of PA’ across three waves of MONICA data in Denmark, and links this ‘pattern of PA’ to measures of changes in weight and body fat distribution, and to all-cause mortality, CVD and CHD mortality. The research questions are whether these ‘PA patterns’ over time show different epidemiological relationships for weight gain and fat distribution and with mortality and CVD/CHD outcomes, compared to single time-point measures of exposure. Specifically, we compared static baseline measures of PA and subsequent health outcomes, specifically (i) PA levels baseline in 1982/3 (time 1) and health outcomes 26 years later and (ii) PA levels at the last (time 3) data collection point (1994) and health outcomes up to 13 years later; compared with (iii) the ‘PA pattern’ that spanned two (time 1 and time 3) or all three (time 1 time 2 time 3) time points. We expected that a more proximal (to outcome) exposure and a measure of exposure pattern to have stronger predictive value than a single more distal measure.

## Methods

The data source for this analysis was the Danish MONICA Study [17], an international study conducted under the auspices of the World Health Organisation (WHO) to monitor trends in and determinants of mortality from CVD. Data were collected over three waves with surveys in 1982–3 (time 1), 1987–8 (time 2) and 1993–4 (time 3). In 1982, a total 4807 people, born either 1922, 1932, 1942 or 1952 were selected from the Central Person Register as a sex and age stratified random sample of the population in the Western part of the Copenhagen area [18]. Subjects not born in Denmark were excluded (*n* = 226). The remaining 4581 subjects were found to be reasonably representative of the total Danish population with respect to sex, age, educational level, occupation, and housing, but persons employed in agriculture, horticulture, or fishery and self-employed and unskilled workers were slightly underrepresented. This group was invited to participate in a health examination at the Glostrup Hospital undertaken between 1982 and 1984; in total 3608 (79%) participated. Five and 11 years later those still living who had participated in the first survey were re-invited to take part in the follow-up studies [19]. At the first re-examination in 1987–88, 2987 subjects (83%) attended while at the second re-examination in 1993–94, 2656 subjects (74%) attended. In total 3097 attended two or more health examinations. The project was approved by the Ethics Committee for Copenhagen county and is in accordance with the Helsinki II Declaration on Human Rights.

### Physical activity measures

The leisure time PA question used was based on the questionnaire constructed by Saltin and Grimby [20] and asked respondents to report their habitual weekly exercise as (i) none (classified as ‘inactive’), (ii) moderate PA less than 4 h/week (classified as ‘moderately active’), (iii) moderate PA more than 4 h/week and (iv) strenuous, vigorous PA more than 4 h week (classified as ‘highly active’). Although covering only one domain of PA, this measure has previously been shown to be a strong predictor for CVD and mortality and has been validated relating to maximal oxygen uptake [20]. The latter two categories were combined due to small group sizes into a ‘highly active’ category based on volume and intensity. These classifications (‘inactive’, ‘moderately active’ and ‘highly active’) were used for defining levels for the single time point measures of PA at time 1 and time 3.

Additionally, a new combined measure was constructed capturing ‘PA pattern’ over time and reflected the degree of maintenance of PA during the study period. The lowest category, ‘inactive/low maintainers’, included those who were inactive or moderately active at time 1, and inactive at time 3 (and time 2 if they had data for this survey). ‘Active maintainers’ were those who had at least a moderate level of activity at time 1 and high activity at time 3 (and time 2 if they had data for this survey). The ‘mixed PA pattern’ included respondents whose PA did not follow either of these patterns (ie., did not show a stable ‘inactive‘or ‘high activity’ level as defined above).

In addition, two additional measures were used from questions asked about other domains of PA. Specifically, these asked about sport participation (“Do you do any kind of sport or exercise more than one hour per week?” (response categories: yes/no), and the frequency of active travel (“How long do you spend on an average workday walking and biking?” with the respondent estimating the number of minutes). Both sport participation and active travel were only asked at time 1 and time 2. The sport question grouped subjects into those choosing to perform sport and those who did not, as this information could not be separated from the information on leisure time PA. For the current analysis sport level is described as ‘none’ versus ‘any’. The question on active travel asked “About how much time, on a usual day, have you walked or ‘cycled?” To create categories reflective of low, moderate and high in a comparatively high active-travel population [21], we classified respondents into low (0–19 min/day), moderate (20–39 min) and high (40+ mins/day), as other research has estimated Danes cycle to work for three hours per week on average [22]. The ‘sport pattern’ variable was constructed as people not doing any sport at both time 1 and time 2, and the ‘sport maintainer’ category were people reporting sport at both time points, and the remainder, who did sport once at either time 1 or time 2, were categorized as ‘mixed’. Similarly for cycling/walking, those reporting nil or less than 20 min/day at both time points were categorized as ‘low maintainers of active travel’, those reporting 40+ minutes/day at both time points were characterized as ‘high maintainers’, and the remainder were ‘mixed pattern’.

### Health outcomes

#### Anthropometric measures

Anthropometric measurements were taken by a trained nurse in accordance with WHO standards [23]. Body weight was measured to the nearest 0.1 kg using a SECA balance scale, with individuals dressed in light clothing or underwear. Height was measured to the nearest 0.5 cm, with individuals wearing no shoes, feet close together, and head held in the horizontal plane [24]. Waist circumference was measured to the nearest cm midway between lower rib margin and the iliac crest in the horizontal plane. Hip circumference was measured to the nearest centimetre at the point yielding the maximum circumference using a tape measure. Analyses examined the relationship between the PA and BMI (both in BMI units and percentage change from baseline) from time 1 to time 3. We also examined relations with waist- and hip circumference (only assessed at time 2 and time 3). Hip circumferences were recoded into those less than and those greater than 100 cm, as this is a potential indicator of risk [25] and appears to be especially strong for women [26].

#### Mortality

Participants initially free from coronary heart disease (CHD), stroke, and cancer were followed up until 2007 through personal identification numbers at the National Registers of Hospital Discharge and Death Registry. Three outcome variables were used to examine the relationship between the PA measures and mortality: all-cause mortality, death caused by CVD and death from CHD defined according to *International Classification of Diseases (ICD)*, Eighth and Tenth Revisions, ICD-8 codes 390–458 and ICD-10 codes I00-I52, and I60-I99 were used to evaluate death of CVD. For death from CHD ICD-8 codes 410–414 and ICD-10 codes I20-I25 were used. Data on all participants could be retrieved from the registries.

Events were identified by record linkage to the Cause of Death Registry, including information regarding all deaths since January 1943, and the National Patient Registry including information regarding all hospitalizations since 1977 [27]. Events ascertainment was made by review of medical files for participants from the 1914 cohort who were included in 1974 and thus before 1977 [28]. Documentation of the validity of the diagnosis of myocardial infarction (*International Classification of Diseases*, Eighth Revision, code 410) in the National Patient Registry and the Cause of Death Registry has been published earlier [29].

#### Analysis

The analyses examining the relationship between PA and change in body mass index (BMI) and BMI percent, waist circumference (at time 3) used linear regression, with age, gender and education (as a proxy for socioeconomic status) as covariates; logistic regression models were used to assess the relationship with hip circumference, with the adjusted odds ratios reflecting the odds of being in the low risk (>100 cm) group at time 3. Three models were run for each outcome variable: the first used PA measured at time 1, (PA1), the second used PA measured at time 3 (PA3), and the third assessed ‘PA pattern’, measured at time 1 and time 2 and/or 3. PA in all models was entered as a categorical variable with the lowest level of PA as the reference category. Each model excluded those respondents who did not have PA data at least at both time 1 and time 3 (*n* = 588), to ensure that the sample was consistent across the three analyses for each outcome. Identical procedures were used to examine sport at time1 and time 2, and ‘sport participation pattern’, and active travel (walk, cycle) at time1 and time2, and ‘active travel pattern’. The analyses for waist and hip circumference were run including BMI at time 1 and BMI percent gain time 1 to time 3 as recommended in previous research [25].

For the analyses examining relationships with all-cause and CVD cause-specific mortality, Cox proportional hazards models and competing risk analyses respectively, with age at screening as the time of study entry and age at death/censoring as the exit time, were run. Age rather than time-on-study was used as the time scale as previous simulation studies have shown that the former method yields more accurate results because risk estimates are calculated on people of the same age [30]. Time at risk was calculated from time 3 for the PA analyses and time 2 for the sport and active travel analyses because, as stated above, to ensure comparable models only respondents attending these assessments were included in the analyses and therefore all included respondents would have had to have survived at least to those assessments). Respondents who had been diagnosed with CVD prior to baseline as recorded by the National Patient Registry (*n* = 189) were excluded from the mortality analyses in order to minimize reverse causality. Results are expressed in terms of hazard ratios (HR) (for all-cause mortality) and subhazard ratios (SHR) (for cause-specific mortality) of the category of interest compared with the reference category, along with 95% confidence intervals. The comparison group for deaths from all causes were those still alive at the censored time point, and for CVD and CHD deaths, those still alive along with those dying from other causes. As with the analyses of the anthropometric measures, models using the three different measures for PA were run on the same sample of respondents and used the relevant PA measure as a categorical variable.

All analyses were conducted using Stata 13.0 [31]. A threshold of 0.05 for statistical significance was used.

## Results

The baseline sample collected in 1982/3 was comprised of 51.1% males, around a quarter of the sample in four roughly equal age groups (adults 30/31 years, 40/41 years, 50/51 years and 60/61 years in 1983). The samples comprised 3609 adults in 1983, with 2998 in 1988 and 2555 in 1994; of these 2509 had matched data for the PA variables (at least time 1 and time 3), 2966 and 2960 were matched for the sport and walk/bike exposures (time 1 and 2 only) respectively. Almost a quarter of the sample had completed 12 or more years of education.

The data for PA at times 1 and 3, and the ‘PA pattern’ are shown in Table 1. These data were from the matched sample, but did not differ on the prevalence of PA levels, weight or age/gender or education from the full baseline sample in 1982. PA in leisure time showed a slight decline in those reporting ‘none’ across the three time periods. The ‘pattern of PA’ over time indicated that less than 12% remained inactive (‘inactive/low maintainers’), over two thirds showed a ‘mixed pattern’, and 19% remained at least moderately active for four or more hours per week (‘active maintainers’). Sport participation at time 1 and time 2 were similar, with around 32% reporting any sporting activity each week; the ‘pattern’ of sport participation across both time periods showed a much lower prevalence, and 19.0% reporting participating in sport at both data collection periods. Active travel through walking or cycling was asked at time 1 and 2, and showed an increase in the proportion reporting 40 + minutes of active travel between 1983 and 1988 (from 51.6 to 59.1%). The proportion maintaining this high level of active travel at both time points was 37.3%, with a small proportion reporting low active travel at both time points (7.5%).

**Table 1:** Patterns of physical activity, sport, active travel and weight over time (confined to matched samples)

Measure		1983	1988	1994	Patterns of activity or weight change
		Time 1	Time 2	Time 3	
PA in leisure time^a^		%	%	%	%
Level 1	None	23.7	22.8	20.8	
Level 2	Moderate <4 h	53.7	57.9	56.9	
Level 3	Mod or strenuous >4 h	22.6	19.3	22.3	
PA pattern^a^	Inactive/low maintainers				11.6
	Mixed pattern				69.4
	Active maintainers				19.0
Sport participation^b^	% reporting nil	68.3	67.8		
	% reporting any	31.7	32.2		
Sport participation pattern^b^	No sport at time1 & time2				55.1
	Mixed sport reported once				25.9
	Sport both times				19.0
Active travel Walk/bike^c^	<20 mins/d	21.7	15.8		
	20–39 min/d	26.7	25.2		
	40+ mins/d	51.6	59.1		
Active travel pattern^c^	<20mins both times				7.5
	Mixed pattern				55.2
	>40 mins both times				37.3
BMI (μ, sd)^a^		24.4 (3.7)	25.1 (3.9)	25.9 (4.1)	
BMI change time 1 to time 3 μ (sd)^a^					1.5 (2.2)
Percent BMI change time 1 to time 3 μ (sd)^a^					6.3% (9.0)
Mean waist circumference female/male in cm^a^ (sd)		–	78.4/91.4 (12.1)	81.1/93.7 (12.4)	2.8/2.3 (6.1)^d^
Hip circumference female/male in cm (sd)^a^		–	98.1/98.5 (7.3)	99.3/99.3 (8.0)	1.3/0.80 (3.9)^d^

^a^Matched data based on *n* = 2509 with a value for PA time 3 (2388 (95.2%) had data at all three time points)

^b^Matched data based on *n* = 2966 with a value for sport time 1 and time 2

^c^Matched data based on *n* = 2960 with a value for walk/bike time 1 and time 2

^d^Change from time 2 to time 3 as not collected at time 1

BMI, waist and hip circumference data are shown in the lower half of Table 1. There was an increase in BMI over time averaging one and a half BMI units from time 1 to time 3. Waist circumference increased by 2.6 cm (2.8 cm for women and 2.3 cm for men) and hip circumference by just over a centimeter between time 2 and time 3 (1.3 cm for women and 0.80 cm for men).

The relationship between PA and weight change is shown in Tables 2 and 3. Note that sensitivity analyses examining the effect of excluding those who did not have PA data at T2 (but did have data for T1 and T3, *n* = 121) showed the findings below changed little if these people were removed the analyses. There was no relationship between PA at time 1 and subsequent change in BMI or BMI percent but the high PA group at time 3 showed a significantly lower 0.44 BMI unit and 1.6% BMI percent lower gain compared with the inactive group (*p* < 0.01 for both). Similar relationships were seen with the ‘pattern of PA’ where ‘active maintainers’ showed a 0.44 BMI unit and 1.5% percent less BMI gain than the ‘inactive/low maintainers’ (*p* = 0.02).

**Table 2:** Adjusted^a^ BMI change, waist and hip circumference by leisure time physical activity pattern (*n* = 2508)

		Beta coefficients (95% CI)			Adjusted odds ratios
PA measure		BMI gain	BMI gain %	Waist time3 [cm]^b^	Hip circumference
		time1 to time3	time 1 to time 3		>100 cm at time 3^b^
PA time 1 (1982/3)	Inactive	Ref	Ref	Ref	Ref
	Moderately active	−0.10 (−0.31, 0.12)	−0.21 (−1.05, 0.62)	−0.99 (−1.48, −0.49)**	1.07 (0.78, 1.47)
	Highly active	−0.04 (−0.30, 0.22)	−0.14 (−1.16, 0.88)	−1.70 (−2.30, −1.10)**	0.74 (0.50, 1.08)
PA time 3 (1994)	Inactive	Ref	Ref	Ref	Ref
	Moderately active	−0.16 (−0.38, 0.07)	−0.44 (−1.32, 0.43)	−0.34 (−0.86, 0.17)	0.97 (0.70, 1.35)
	Highly active	−0.44 (−0.70, −0.17)**	−1.59 (2.63, −0.54)**	−1.38 (−1.99, −0.76)**	0.80 (0.55, 1.18)
PA pattern	Inactive/low maintainers	Ref	Ref	Ref	Ref
	Mixed pattern	−0.17 (−0.44, 0.10)	−0.42 (−1.50, 0.66)	−0.83 (−1.46, −0.19)*	0.81 (0.53, 1.23)
	Active maintainers	−0.44 (−0.76, −0.12)**	−1.48 (−2.76, −0.21)*	−2.01 (−2.77, −1.26)**	0.63 (0.39, 1.02)

^a^Adjusted for baseline age, sex and education

^b^Adjusting for BMI and time 1 and BMI percent change from time 1 to time 3

*p ≤ 0.05 **p ≤ 0.01

**Table 3:** Adjusted^a^ BMI change, waist and hip circumference by sport (*n* = 2419) and active travel pattern (*n* = 2416)

PA Measure		Beta coefficients			Adjusted odds ratios
		BMI gain time1 to time3	BMI gain % time1 to time3	Waist [cm] time3^b^	Hip circumference > 100 cm at time 3^b^
Sport time1 (1982/3)	None	Ref	Ref	Ref	Ref
	Any	0.10 (−0.09, 0.28)	0.29 (−0.44, 1.02)	−0.88 (−1.32, −0.44)**	0.82 (0.63, 1.07)
Sport time2 (1988)	None	Ref	Ref	Ref	Ref
	Any	−0.10 (−0.28, 0.09)	−0.21 (−0.93, 0.51)	−0.90 (−1.33, −0.46)**	0.80 (0.62, 1.04)
Sport pattern	No sport at time1 & 2	Ref	Ref	Ref	Ref
	Mixed sport reported once	−0.06 (−0.15, 0.26)	0.33 (−0.48, 1.14)	−0.81 (−1.30, −0.32)**	0.92 (0.69, 1.23)
	Sport maintainers at time1 & time2	−0.20 (−0.25, 0.21)	−0.04 (−0.94, 0.85)	−1.23 (−1.77, −0.69)**	0.72 (0.52, 1.00)*
Active travel time1^c^ (1982/3)	40+ mins	Ref	Ref	Ref	Ref
	20–39 min	−0.07 (−0.28, 0.13)	−0.35 (−1.16, 0.45)	−0.22 (−0.71, 0.27)	0.98 (0.73, 1.31)
	0–19 min	−0.11 (−0.34, 0.11)	−0.43 (−1.31, 0.45)	−0.08 (−0.61, 0.46)	1.01 (0.73, 1.38)
Active travel time2§ (1988)	40+ mins	Ref	Ref	Ref	Ref
	20–39 min	0.13 (−0.08, 0.33)	0.46 (−0.35, 1.27)	0.32 (−0.17, 0.80)	0.73 (0.54, 0.98)*
	0–19 min	0.25 (0.01, 0.50)	0.82 (−0.16, 1.81)	0.44 (−0.16, 1.04)	1.10 (0.77, 1.57)
Active travel pattern§	40+ mins maintainers	Ref	Ref	Ref	Ref
	Mixed pattern	−0.06 (−0.25, 0.12)	−0.37 (−1.09, 0.36)	0.01 (−0.43, 0.44)	0.85 (0.66, 1.11)
	0–19 min maintainers	0.03 (−0.39, 0.33)	−0.20 (−1.61, 1.21)	0.79 (−0.07, 1.64)	1.33 (0.82, 2.17)

^a^Adjusted for baseline age, sex and education

^b^Adjusting for BMI and time 1 and BMI percent change from time 1 to time 3

^c^For active travel, reference category was 40+ mins, given the low number in the low active travel (0–19 min maintainers, *n* = 221) category

*p* ≤ 0.05 **p ≤ 0.01

The strongest associations among the anthropometric measures were with waist circumference measured at time 3. Waist circumference was significantly lower for the ‘moderate’ and ‘high’ PA groups at time 1 and at time 3 compared with the inactive or low PA group, up to 1.70 cm (*p* < 0.01). There was an even stronger relationship (by almost 18%) for the ‘pattern of PA’, where ‘active maintainers’ had on average 2.0 cm lower waist circumference than ‘inactive/low maintainers’ (p < 0.01). The adjusted odds ratio of an increased hip measurement at time 3 is shown in the far right hand column, and indicates levels of PA were unrelated to the odds of having a hip circumference greater than 100 cm, irrespective of whether the exposure was single or two time-points.

Sport was not related to BMI gain or percent BMI change (Table 3) for the single or two time-point exposures. Sport at time 1 and time 2 and the patterns of ‘mixed sport’ and ‘maintained sport’ were associated with lower waist circumference at time 3 (compared to ‘no sport’), with ‘sport maintainers’ pattern showing the strongest effect (−1.23 cm, *p* < 0.01) 27% larger than that for the single time point measures. ‘Sport maintainers’ also showed a 28% reduced odds of having a hip circumference greater than 100 cm at time 3 (*p* = 0.05) whereas the single time point measures showed no significant effect. Active travel was not related to BMI change or waist circumference for the single or two time-point exposures, and the odds of having a hip circumference > 100 cm were only significantly lower for active travel at time 2 (20–39 min vs 40+ min, OR = 0.73, *p* = 0.04).

Table 4 shows the mortality risk for each of the PA measures, both the static time 1 and time 3 measures, the ‘pattern of PA’, and Table 5 shows these relationships for sport participation and active travel. All models were examined both analytically and graphically for proportional hazards and the assumption was met in all cases [32]. Risk of all-cause mortality wcat time 3 (the ‘highly active’ group showed a 57% reduction) and the ‘PA pattern’ exposure, (‘active maintainers’ showed a 59% reduction (HR 0.41; 95% CI 0.28–0.59)). CVD deaths were significantly reduced in the ‘highly active’ group at PA at time 3 (43% risk reduction, (HR 0.57; 95% CI 0.35–0.93)) but not time 1; ‘active maintainers’ had a similar hazard ratio (HR 0.61; 95% CI 0.33–1.15) but it did not reach statistically significance. The number of CHD (ischaemic heart disease) deaths was small (*n* = 69); only the ‘active maintainers’ in the ‘PA pattern’ exposure showed risk a reduction of 62% (HR 0.38; 95% CI 0.15–0.96), and none of the single time point measures.

**Table 4:** Risk of death^a^ by pattern of physical activity adjusted for age, sex and education (*n* = 2412)

		Adjusted hazard ratios (95% CI)	Adjusted subhazard ratios (95% CI)	
		All-cause mortality	CVD deaths	CHD deaths
		(445 deaths)	(185 deaths)	(69 deaths)
PA time1 (1982/3)	Inactive^b^	1.0	1.0	1.0
	Moderately active	0.68 (0.54–0.85)**	0.89 (0.62–1.28)	0.88 (0.48–1.62)
	Highly active	0.65 (0.49–0.85)**	0.73 (0.47–1.15)	0.83 (0.40–1.72)
PA time 3 (1994)	Inactive^b^	1.0	1.0	1.0
	Moderately active	0.62 (0.49–0.78)**	0.87 (0.60–1.27)	0.58 (0.33–1.03)
	Highly active	0.43 (0.32–0.58)**	0.57 (0.35–0.93)*	0.53 (0.26–1.09)
PA pattern	Inactive/low maintainers^b^	1.00	1.0	1.0
	Mixed pattern	0.66 (0.50–0.87)**	1.08 (0.66–1.77)	0.59 (0.30–1.18)
	Active maintainers	0.41 (0.28–0.59)**	0.61 (0.33–1.15)	0.38 (0.15–0.96)*

^a^ Excludes those with baseline reported CVD

^b^ Reference category

*p ≤ 0.05 **p ≤ 0.01

**Table 5:** Risk of death^a^ by patterns of sport (*n* = 2834) and active travel pattern (*n* = 2829) adjusted for age, sex and education

		Adjusted hazard ratios (95% CI)	Adjusted subhazard ratios (95% CI)	
PA measure		All-cause mortality	CVD deaths	CHD deaths
		(668 deaths)	(290 deaths)	(125 deaths)
Sport time1 (1982/3)	None^c^	1.0	1.0	1.0
	Any	0.79 (0.66, 0.95)*	0.50 (0.36, 0.69)**	0.44 (0.26, 0.74)** .
Sport time2 (1988)	None^c^	1.0	1.0	1.0
	Any	0.66 (0.55, 0.79)**	0.57 (0.43, 0.77)**	0.51 (0.32, 0.83)**
Sport pattern	No sport at time1 & time2^c^	1.0	1.0	1.0
	Mixed sport reported once	0.80 (0.67, 0.97)*	0.78 (0.56, 1.04)	0.74 (0.47, 1.15)
	Sport maintainers at time1 & time2	0.62 (0.49, 0.79)**	0.33 (0.20, 0.53)**	0.25 (0.11, 0.58)**
		All-cause mortality	CVD deaths	CHD deaths
		(664 deaths)	(291 deaths)	(125 deaths)
Active travel time1^b^ (1982/3)	40+ mins^c^	1.0	1.0	1.0
	20–39 min	0.96 (0.80, 1.16)	0.97 (0.73, 1.29)	0.88 (0.57, 1.37)
	0–19 min	1.11 (0.92, 1.34)	1.13 (0.85, 1.50)	0.75 (0.47, 1.19)
Active travel time2^b^ (1988)	40+ mins^c^	1.0	1.0	1.0
	20–39 min	1.09 (0.89, 1.32)	0.98 (0.72, 1.32)	1.06 (0.67, 1.66)
	0–19 min	1.44 (1.17, 1.78)**	1.19 (0.86, 1.63)	1.07 (0.65, 1.77)
Active travel pattern^b^	40+ mins maintainers^c^	1.0	1.0	1.0
	Mixed pattern	1.06 (0.90, 1.25)	1.01 (0.79, 1.29)	0.95 (0.66, 1.37)
	0–19 min maintainers	1.42 (1.07, 1.88)*	1.30 (0.85, 1.97)	1.10 (0.58, 2.11)

^a ^Excludes those with baseline reported CVD

^b ^For active travel, reference category was 40+ mins, given the small numbers in the low active travel (0–19 min maintainers) category

^c ^Reference category

*p ≤ 0.05 **p ≤ 0.01

Table 5 shows the relationships between sport and active travel and subsequent health outcomes. ‘Any sport’ at time 1 or time 2 (vs ‘none’) lowers subsequent risk of all-cause, CVD and CHD mortality. However, larger risk reductions were noted for the ‘sport pattern’ of maintaining sport on both occasions (compared no sport on both occasions): 38% for all-cause (HR 0.62; 95% CI 0.49–0.79), 67% for CVD (HR 0.33; 95% CI 0.20–0.53) and 75% for CHD (HR 0.25; 95% CI 0.11–0.58) mortality compared to smaller protective benefits of sport at time 2 alone (34%, 43% and 49% risk reductions respectively). The ‘mixed sport pattern’ did not show a stronger effect compared to the single time-point exposures. Only the lowest active travel group at time 2, and the lowest ‘pattern of active travel’ categories showed significant but similar increased risk for all-cause mortality (44% (HR 1.44; 95% CI 1.17–1.78) and 42% (HR 1.42; 95% CI 1.07–1.88) respectively).

## Discussion

This paper proposes potential benefits of characterizing PA exposure as a ‘pattern over time’, especially given the variation in within-individual PA within an individual over time. The benefit was seen across a number of the examined endpoints including overall risk of premature death, risk of CVD and CHD as well as development of obesity and healthy fat patterning. There is known under-estimation of risk using PA self-report measurements [33], and a sustained behavioural pattern may better reflect true exposure. Describing ‘PA patterns’ rather than static and/or single point measures might reduce misclassification bias, and still include behaviours most proximal to the outcomes of interest, as some of the cardio-protective effects of PA may be acute [15, 34].

In this analysis of PA behaviours among Danish adults assessed over 11 years, just less than one-fifth of the cohort maintained moderate to high PA levels at both time points, a prevalence lower than at any single observation in this study. In general, irrespective of the measure, PA levels were not strongly associated with subsequent weight change. The fact that there was no relationship between baseline activity level PA (time 1) and subsequent weight gain may be due to reverse causality, as earlier weight change may precede changes in PA, rather than activity being a predictor of obesity development, as also suggested by others [35]. Other Danish cohort data has shown lack of a relationship between leisure time PA and 10-year subsequent waist measurement or obesity, but did show a small effect for sports participation [36].

Nonetheless, there were significant associations with subsequent development in fat distribution, as assessed by waist circumference at time 3, which in addition showed a dose response relationship across all measures of PA that was evident maintained even accounting for concurrent changes in weight. The strongest relationships were seen for the ‘PA pattern’ and waist circumference, where ‘active maintainers’ had two centimeters lower measures compared to the ‘inactive maintainers’ when adjusted for change in BMI over time. Whilst it is difficult to compare effect sizes with those from previous research because of different operationalization of PA groups, follow-up period and included covariates, the difference between those in the ‘active maintainers’ and ‘no/low active groups’ was broadly in the ranges reported elsewhere [10, 12]. Interestingly, the finding for waist circumference was in the presence of a smaller effect on BMI, suggesting that the distribution of weight may be independently shifted towards a healthier distribution of less abdominal adiposity for those with a sustained PA and those increasing sports and active travel [37].

Previous research has repeatedly shown associations between PA and weight change when changes in activity and weight were measured concurrently, including one study which considered PA at multiple time points using repeat measures regression to demonstrate that around an hour per day of moderate PA was associated with the lowest weight gain [38]. However, this kind of repeat-measures analysis includes regression of data from all time points, but is not the same as the ‘behavioural patterning’ described here. One study which did include both baseline only and PA pattern analyses appeared to show a stronger effect of PA on weight between the single-point and multiple time-point analyses, however the comparisons were between those who increased or decreased their PA from one time-point to the next compared with those who did not, irrespective of level (ie., constantly high or low) making the results difficult to compare with the current study [11]. Hence this study has quantified the effect of a particular and clinically relevant characterization of PA over time, rather than only account for within-individual change, and additionally compared it to the more frequently used single point analysis. Notably though, the more proximal single point PA measure analyses also showed strong associations with outcomes demonstrating the value of both compared with a single, distal measure.

In the present study ‘PA maintainers’ showed lower CHD risk than the baseline PA measure alone, although the risk reduction for all-cause mortality was similar to ‘high PA’ assessed at time 3 alone. Previous Danish research has shown PA protects against all-cause and CVD mortality, with the latter showing relative risk reductions of 29% for moderate activity, and 44% for high PA levels [39], while a British sample of older (40–59 years) men showed risk reductions of 59% for all-cause and 63% for CVD-mortality for ‘light/moderate active’ vs. ‘inactive/occasional active’ maintainers [40]; the present did not show such effects in relation to CVD endpoints except for the PA at time 3. Further, the survival benefit conferred by PA of around 3–5 years [41] may also be an underestimate for the same reasons. The small number of deaths for CVD (n-185) and CHD (*n* = 69) may have contributed to the lower number of statistically significant findings despite trends mostly in the expected direction.

Sport participation measured at a single point in time in several studies, has been shown to be independently associated with mortality [42]. Our findings suggest that sport might be better characterized as a pattern, with only 15.6% participating in sport at the two time points observed. The pattern of ‘maintained sport participation’ was more strongly associated with CHD outcomes than either time 1 or time 2 sport participation in isolation. However, genetic factors, and possibly genetic pleiotropic effects, could be effect modifiers of the relationship between sport and mortality, and that should be investigated in relation to PA patterns and survival risk where such genetic information is available [43]. For active travel, which has shown protective effects in other populations [44, 45] there were no significant relationships with CVD or CHD mortality, only all-cause; but given the high rates of walking and cycling in and around Copenhagen [46], the unexposed group of non-active commuters was small in the current study, reducing variation in this exposure measure and making it more difficult to detect differences.

### Strengths and limitations

A strength of this study is that it has proposed an arguably stronger operationalization of PA exposure for epidemiological studies, and tested it against more conventionally used measures. This study had repeat measures to characterize exposure across PA domains, including sport and active travel. The MONICA study also has a long follow-up time allowing for robust mortality analyses. One potential limitation of this study was not to adjust weight change for dietary measures and other potential confounders and covariates. However, the research purpose of this paper was not to provide fully adjusted independent estimates of risk for PA, but to compare across single versus ‘PA pattern’ measures; it is assumed that dietary and other contributions to risk are non-differential, and would not confound ‘PA patterns’ notably more than single-point PA estimates. It is also the case that in an observational epidemiological setting, a third variable may explain both why some individuals are physically active and have reduced risk of death, and therefore the observed relationships are not conclusive proof of causality. Another limitation is that sport and active travel were measured at two time points only (time 1 and time 2) making comparisons with the analyses for leisure time PA more difficult, and not having a PA measure in these domains more proximal to the health outcome. Further, as the sample in the PA analysis by definition must have survived to time 3, they may constitute a healthier group than those in the sport and active travel analyses and therefore comparisons across the measures should be made with caution. Finally, the 'mixed' pattern did not distinguish between those who increased or decreased their activity from the first to second time point perhaps obscuring these effects. Further, including moderate maintainers in the mixed pattern could have masked effects of a more internally consistent mixed pattern of active travel. However, the sample sizes, especially in the cause-specific mortality analyses, were too low to further divide this group.

## Conclusion

This paper presents a new hypothesis, that the concept of long term behavioural ‘patterning of PA’ that may extend usual epidemiological practice (as shown in Additional file 1), where most studies have assessed PA exposure at only one time point, followed by subsequent health outcomes. We found ‘PA pattern’ has similar effect sizes for all-cause and CVD mortality as a single-but-proximal measure, but stronger for CHD mortality and waist circumference. BMI relationships are similar for proximal and pattern exposures. Sport pattern is consistently stronger than both single time-point measures. This method may improve exposure measurement compared to just assessing static exposure to PA at a baseline assessment and may prove to be a methodological advance for population studies at the intersection between behavioural expertise and epidemiology. Longitudinal studies could conceptualize and re-examine PA behavioural patterning in more fine-grained detail, and refine the more subtle population risks of individuals adopting, maintaining and relapsing from PA over multiple data points as potentially better predictors of health outcomes [47]. Further exploration of interaction of these patterns with concurrent sedentary behaviour time and concurrent weight status could improve the evidence base for the life course benefits of PA on health.

## Additional file

Additional file 1: Bibliometric analysis of publications: what is the rate of single PA exposure measures in epidemiological studies? Bibliometric analysis of publications 1975–2000 and 2014–2016. (DOCX 15 kb)

## Abbreviations

BMI: Body Mass index
CHD: Coronary Heart Disease
CI: Confidence interval
CVD: Cardiovascular Disease
HR: Hazard ratio
ICD: International Classification of Diseases
MONICA: MONItoring Trends and Determinants in CArdiovascular Disease
PA: Physical activity
sd: Standard deviation
SHR: Subhazard ratio
WHO: World Health Organisation
