# Supplementary material for: Physical activity, obesity and mortality: does pattern of physical activity have stronger epidemiological associations?
Source: BMC Public Health. 2017 Oct 5;17:788. doi: 10.1186/s12889-017-4806-6 (PMC5629749; doi:10.1186/s12889-017-4806-6)
Supplement: Additional file 1: — Bibliometric analysis of publications: what is the rate of single PA exposure measures in epidemiological studies? Bibliometric analysis of publications 1975–2000 and 2014–2016. (DOCX 15 kb) [file 12889_2017_4806_MOESM1_ESM.docx]

**Additional File 1: Bibliometric analysis of publications: what is the rate of single PA exposure measures in epidemiological studies?**

The purpose of this analysis was to estimate the rates of ‘single exposure measurements’ of physical activity or fitness in epidemiological studies. We search term for identifying exposure from the titles of papers using the terms 'physical activity' or 'fitness' and combined them with mortality or incidence. Note that this was not a complete review of all studies in all databases, but should not be a systematically biased sample, and hence provides a comprehensive estimate of the proportion of single PA exposure measurements in physical activity (PA) epidemiological studies.

The initial period reviewed was between 1975 to 2000 where we identified n=148 papers using the following search terms and Scopus database: [PA/fitness] and [cardio* OR CVD OR mortality OR incidence] in the *title* of the paper.  Papers were reviewed and coded by two authors (AB, VR), and agreement was 0.97, which was reconciled by discussion. We excluded 57 (reviews, commentaries, no abstract), which left 91 studies that we examined for PA/fitness and mortality/incidence outcomes for CVD and all-cause mortality. Of these, 8 studies (9%) had two or more measures of PA/fitness, and 83 (91%) had only single time point measure of PA/fitness.

For the recent period of publications between 2014 and 2016, we used exactly the same search strategy. In this period, 111 studies examined PA and health outcomes and only eight studies, had two or more measures of PA/fitness, while 103 (93%) had a single time point measure of PA/fitness.

These findings point to a high rate of single physical activity exposure measurements, with no difference between the earlier period (91%, 95%CI 84-95%), and the more recent period (93%, 95%CI 88-98%). Examination of the studies that used two or more PA measures* suggested serial measurement was not always intentional, but data had opportunistically been collected twice. Only a very few studies specifically addressed change in physical activity levels, conceptually closer to the ‘PA pattern’ proposed in this paper.

** Tthese 2+ PA exposure studies are listed in Appendix A online for the early (1975-2000) and recent (2014-2016) periods.*

References for Additional File 1

1. Bijnen FC, Feskens EJ, Caspersen CJ, Nagelkerke N, Mosterd WL, Kromhout D: Baseline and previous physical activity in relation to mortality in elderly men: the Zutphen Elderly Study. *Am J Epidemiol* 1999, 150(12):1289-1296.

2. Blair SN, Kohl HW, 3rd, Barlow CE, Paffenbarger RS, Jr., Gibbons LW, Macera CA: Changes in physical fitness and all-cause mortality. A prospective study of healthy and unhealthy men. *JAMA* 1995, 273(14):1093-1098.

3. Dorans KS, Massa J, Chitnis T, Ascherio A, Munger KL: Physical activity and the incidence of multiple sclerosis. *Neurology* 2016, 87(17):1770-1776.

4. Erikssen G, Liestol K, Bjornholt J, Thaulow E, Sandvik L, Erikssen J: Changes in physical fitness and changes in mortality. *Lancet* 1998, 352(9130):759-762.

5. Esteban C, Garcia-Gutierrez S, Legarreta MJ, Anton-Ladislao A, Gonzalez N, Lafuente I, Fernandez de Larrea N, Vidal S, Bare M, Quintana JM *et al*: One-year Mortality in COPD After an Exacerbation: The Effect of Physical Activity Changes During the Event. *COPD* 2016, 13(6):718-725.

6. Etemadi A, Abnet CC, Kamangar F, Islami F, Khademi H, Pourshams A, Poustchi H, Bagheri M, Sohrabpour AA, Aliasgar A *et al*: Impact of body size and physical activity during adolescence and adult life on overall and cause-specific mortality in a large cohort study from Iran. *Eur J Epidemiol* 2014, 29(2):95-109.

7. Jefferis BJ, Whincup PH, Lennon LT, Papacosta O, Goya Wannamethee S: Physical activity in older men: longitudinal associations with inflammatory and hemostatic biomarkers, N-terminal pro-brain natriuretic peptide, and onset of coronary heart disease and mortality. *J Am Geriatr Soc* 2014, 62(4):599-606.

8. Paffenbarger RS, Jr., Hyde RT, Wing AL, Lee IM, Jung DL, Kampert JB: The association of changes in physical-activity level and other lifestyle characteristics with mortality among men. *N Engl J Med* 1993, 328(8):538-545.

9. Rosenberg L, Palmer JR, Bethea TN, Ban Y, Kipping-Ruane K, Adams-Campbell LL: A prospective study of physical activity and breast cancer incidence in African-American women. *Cancer Epidemiol Biomarkers Prev* 2014, 23(11):2522-2531.

10. Sherman SE, D'Agostino RB, Silbershatz H, Kannel WB: Comparison of past versus recent physical activity in the prevention of premature death and coronary artery disease. *Am Heart J* 1999, 138(5 Pt 1):900-907.

11. Steffen-Batey L, Nichaman MZ, Goff DC, Jr., Frankowski RF, Hanis CL, Ramsey DJ, Labarthe DR: Change in level of physical activity and risk of all-cause mortality or reinfarction: The Corpus Christi Heart Project. *Circulation* 2000, 102(18):2204-2209.

12. Stenholm S, Koster A, Valkeinen H, Patel KV, Bandinelli S, Guralnik JM, Ferrucci L: Association of Physical Activity History With Physical Function and Mortality in Old Age. *J Gerontol A Biol Sci Med Sci* 2016, 71(4):496-501.

13. Vaes AW, Garcia-Aymerich J, Marott JL, Benet M, Groenen MT, Schnohr P, Franssen FM, Vestbo J, Wouters EF, Lange P *et al*: Changes in physical activity and all-cause mortality in COPD. *Eur Respir J* 2014, 44(5):1199-1209.

14. Wannamethee SG, Shaper AG, Walker M: Changes in physical activity, mortality, and incidence of coronary heart disease in older men. *Lancet* 1998, 351(9116):1603-1608.

15. Wannamethee SG, Shaper AG, Walker M: Physical activity and mortality in older men with diagnosed coronary heart disease. *Circulation* 2000, 102(12):1358-1363.

16. Zethelius B, Gudbjornsdottir S, Eliasson B, Eeg-Olofsson K, Cederholm J, Swedish National Diabetes R: Level of physical activity associated with risk of cardiovascular diseases and mortality in patients with type-2 diabetes: report from the Swedish National Diabetes Register. *Eur J Prev Cardiol* 2014, 21(2):244-251.
